# Health Status of Great Frigatebirds (*Fregata minor*) Determined by Haematology, Biochemistry, Blood Gases, and Physical Examination

**DOI:** 10.1093/conphys/coy034

**Published:** 2018-07-02

**Authors:** Carlos A Valle, Catalina Ulloa, Diane Deresienski, Cristina Regalado, Juan-Pablo Muñoz-Pérez, Juan Garcia, Britta Denise Hardesty, Alice Skehel, Gregory A Lewbart

**Affiliations:** 1 Universidad San Francisco de Quito USFQ, Colegio de Ciencias Biológicas y Ambientales, Extensión Galápagos, Galápagos Casilla Postal 17-1200-841, Quito, Ecuador; 2 Department of Clinical Sciences, College of Veterinary Medicine, North Carolina State University,1060 William Moore Drive, Raleigh, NC, USA; 3 Galapagos Science Center, Universidad San Francisco de Quito (USFQ) & The University of North Carolina (UNC) at Chapel Hill, San Cristóbal Island, Galápagos, Ecuador; 4 Dirección Parque Nacional Galápagos, Unidad Técnica Operativa San Cristóbal, Galápagos, Ecuador; 5 Commonwealth Scientific and Industrial Research Organization, Hobart, Tasmania, Australia

**Keywords:** Biochemistry, blood gases, *Fregata minor*, Galápagos Islands, health status, haematology, San Cristóbal

## Abstract

The great frigatebird, *Fregata minor*, is a widely distributed seabird native to the Galápagos archipelago. Haematology and blood chemistry parameters have been published for this species but not from the San Cristóbal and North Seymour great frigatebird breeding colonies. Analyses were run on blood samples drawn from 25 great frigatebirds captured by hand at their nests at Punta Pitt on San Cristóbal Island and 30 birds on North Seymour Island, Galápagos Islands. A portable blood analyser (iSTAT) was used to obtain near immediate field results for pH, pO_2_, pCO_2_, TCO_2_, HCO_3^−^_, haematocrit (Hct), haemoglobin (Hb), sodium (Na), potassium (K), chloride (Cl), ionized calcium (iCa), creatinine, urea nitrogen, anion gap and glucose. Blood lactate was measured using a portable Lactate Plus™ analyser. Average heart rate, respiratory rate, body weight, body temperature, biochemistry and haematology parameters were comparable to healthy individuals of other Fregatidae. The reported results provide baseline data that can be used for comparisons among populations and in detecting changes in health status among Galápagos great frigatebirds.

## Teaser

This study presents important morphometric and health parameters of 55 great frigatebirds from two locations on the Galápagos archipelago. All of the animals were robust, clinically healthy and behaving normally. This data adds to baseline health assessment values and provides reference blood parameter intervals.

## Introduction

The great frigatebird (*Fregata minor*) is a member of the Suliformes in the family Fregatidae and is one of the most widely distributed members of the five-species family; it occurs throughout the Galápagos archipelago (Valle *et al.*, 2006; Padilla *et al.*, 2006). While there are published reports on haematology and blood chemistry values for this species (Work, 1996; Padilla *et al.*, 2006) ours is the first from San Cristóbal Island and Seymour Norte, and only the second from the Galápagos archipelago.

The Galápagos Islands, located hundreds of kilometres from mainland Ecuador, are vulnerable to the threats from introduced pathogens and parasites (Edgar *et al.*, 2008). The more baseline information we have from clinically normal animals, the better our understanding of any changes that might occur in a species’ health status. In this study, we provide a detailed account of over 30 anatomical, physiological, and blood-specific values that are widely used to evaluate the health of wild seabirds (Work, 1996; Padilla *et al.*, 2006; Ratliff *et al.*, 2017). The sample size is robust (*n* = 55) and represents an accurate accounting of this species on two Galápagos islands.

While there is currently little evidence for introduced diseases impacting *F. minor*, other Galápagos avian species, including members of the Fregatidae, have been affected by introduced pathogens (Gottdenker *et al*., 2008; Levin and Parker, 2012; Bastien *et al.*, 2014). To date, very little work has been done to assess the health of *F. minor* in the Galápagos, with one paper reporting seven of 24 birds (29.2%) positive for a *Haemoproteus*-like blood parasite (Padilla *et al.*, 2006) and one reporting three species of ectoparasitic lice (*Pectinopygus gracillicornis, Colpocephalum spineum, Fregatiella aurifascieta*) with a mean prevalence of 97.2%. 91.6% and 34.6%, respectively, in the 109 birds sampled (Rivera-Parra *et al.*, 2014). *Haemoproteus iwa* has been reported in Western Indian Ocean frigatebirds (Bastien *et al.*, 2014) and in 5–20% of Galápagos great frigatebirds from five different islands, which included North Seymour, but not San Cristóbal (Levin and Parker, 2012). Frigatebirds are unique among seabirds because they commonly harbour hemoparasites (Levin and Parker, 2012; Merino *et al.*, 2012) and the clinical significance is largely unknown.

One objective for this and other health assessments is to monitor how wild animals, like seabirds, respond to and are impacted by climate change (Altizer *et al.*, 2013). To accomplish this, a baseline of normal, or at least health parameter values from a clinically robust population, is crucial. Therefore, it is important to assess the health of wild species, especially if the population in question has never been surveyed, or not surveyed in many years.

To assess the health status of great frigatebirds, and to compare it with other studies, we used the iSTAT machine, a handheld electronic device able to measure a wide variety of blood gas and chemistry parameters with only a few drops (95 μl) of whole, non-coagulated blood. The iSTAT was selected for a variety of reasons. First, it is very portable, sturdy and easy to use in the field. Secondly, since our research team has access to several of the devices, we are able to keep research costs low, and have valuable backup equipment in the field. Finally, the veterinary literature is rich with studies that have utilized the iSTAT for avian health assessment from a variety of species (Steinmetz *et al.*, 2007; Harms and Harms, 2012; Rettenmund *et al.*, 2014; Harter *et al.*, 2015; Ratliff *et al.*, 2014, 2017).

## Materials and methods

This study was performed as part of a population health assessment authorized by the Galapagos National Park Service (permit no. PC-57-16 and no. PC-59-17 to C.A.V.) and approved by the Universidad San Francisco de Quito ethics and animal handling protocol. All handling and sampling procedures were consistent with standard vertebrate protocols and veterinary practices.

### Study area

Data were collected at Punta Pitt, San Cristóbal Island (0° 41′ 59″ S; 89° 15′ 09″ W) and North Seymour Island (0° 23′ 30″ S, 90° 17′ 0″ W) from two of the 13 great frigatebird breeding colonies in the Galápagos archipelago. The North Seymour colony has about 130 breeding pairs and the colony at Punta Pitt by the islet is a relatively new colony of about 60 breeding pairs (C.A.V. unpublished).

### Sampling

In June 2016 (Punta Pitt) and June 2017 (North Seymour), 55 adult great frigatebirds were captured one at a time, examined and sampled before the next bird was selected. Before blood sampling, the animals were weighed, standard measurements were taken, and body temperature, heart rate, and respiratory rate recorded (Table 1). To minimize the potential effects of handling on blood parameters, samples were obtained as soon as safely possible and in most cases within 10 min of capture. All of the birds were deemed clinically healthy based on their behaviour, response to handling and physical examination by veterinarians.

**Table 1:** Summary of morphological and physiological measurements with time to obtain blood samples, reported in mean, standard deviation and range for great frigatebirds (*Fregata minor*); 17 males and eight females from Punta Pitt and 18 males and 12 females from North Seymour Island

Sites	Parameter	Male	Female
Punta Pitt (2016)	Bill length (mm)	80.46 ± 7.78 (55–89)	97.61 ± 4.17 (93–105)
	Bill depth (mm)	19.96 ± 2.01 (15–23)	22.86 ± 5.14 (18–34)
	Bill width (mm)	24.65 ± 1.20 (22–27)	25.78 ± 1.11 (24–27)
	Wing length (cm)	57.75 ± 3.75 (46–61)	60.33 ± 1.58 (57–63)
	Tarsus length (cm)	2.30 ± 0.31 (1.8–3)	2.35 ± 0.22 (1.9–2.7)
	Weight (kg)	1.18 ± 0.14 (0.9–1.5)	1.47 ± 0.12 (1.2–1.6)
	Body Temperature (°C)	38.88 ± 0.93 (37–40)	38.78 ± 1.33 (36–40)
	Heart rate (beats/min^−1^)	223.5 ± 87.3 (110–500)	200 ± 50.42 (130–270)
	Respiratory rate (b/min)	32.35 ± 7.52 (20–50)	30 ± 7.55 (20–40)
	Sample time (min)	7.26 ± 2.63 (3–12)	6.12 ± 2.35 (2–10)
	Handling time (min)	12.21 ± 3.64 (7–19)	10.75 ± 2.12 (8–14)
North Seymour (2017)	Bill length (mm)	98.28 ± 4.02 (92–106)	109.15 ± 3.96 (101–115)
	Bill depth (mm)	23.50 ± 1.89 (20–27)	24.85 ± 1.38 (22–26)
	Bill width (mm)	26.7 ± 1.78 (22–29)	28.66 ± 1.84 (26–33)
	Wing length (cm)	58.16 ± 4.64 (42–62)	61.14 ± 1.49 (59–64)
	Tarsus length (cm)	2.35 ± 0.49 (0.1–0.4)	2.28 ± 0.28 (1.7–2.6)
	Weight (kg)	1.19 ± 0.09 (1–1.3)	1.49 ± 0.12 (1.2–1.6)
	Body Temperature (°C)	39.62 ± 0.64 (38–40)	39.66 ± 0.9 (37–41)
	Heart rate (beats/min^−1^)	197.11 ± 28.14 (148–248)	194 ± 21.94 (152–224)
	Respiratory rate (b/min)	24.44 ± 6.27 (13–36)	26 ± 5.25 (20–32)
	Sample time (min)	5.52 ± 2.78 (3–14)	4.83 ± 1.74 (3–8)
	Handling time (min)	13.77 ± 5.54 (8–29)	12.58 ± 1.97 (10–17)

### Blood sample collection and handling

Brachial vein venipuncture was performed using a heparinized 22-gauge needle and 3.0 ml syringe to collect up to 1.0 ml of blood per bird. The blood was then immediately divided into sub-samples, which were either used for instant analysis with portable blood chemistry and lactate analysers or stored on ice in sterile plastic vials within 10 min of sample collection for haematology and future analyses. Blood films were made immediately and fixed and stained with Diff Quik (Jorgenson Laboratories, Loveland, CO, 80538 USA) ~2 weeks after sampling. Due to the remote nature of the field sites, manual haematocrit levels could not be accurately recorded for all samples, though iSTAT calculated haematocrits for all were recorded. The stained blood films were used for the 100-cell WBC differentials and for estimated WBC counts (Work, 1996), modified from a 40× to 100× objective (Newman *et al.*, 1997).

### Blood gas and biochemistry parameters

The blood gas, electrolyte and biochemistry results were obtained using an iSTAT Portable Clinical Analyser (Heska Corporation, Loveland, Colorado, USA 80538) with CG8+ and CHEM8+ cartridges. The iSTAT is a portable, handheld, battery-operated electronic device with the ability to measure a wide variety of blood gas, chemistry and basic haematology parameters with only a few drops (95 μl) of whole, non-coagulated blood. The unit weighs about 600 g, is powered by two 9-V batteries and utilizes cartridges that can perform a variety of specific tests utilizing embedded electronic sensors. In most cases, results are available within 2 min. The unit has ample memory but can store up to 1000 sample results. The following parameters were measured and recorded: pH, pO_2_, pCO_2_, TCO_2_, HCO_3^−^_, Hct, Hb, Na, K, Cl, iCa, Creatinine, Urea Nitrogen, Anion Gap and glucose. The device analysed the blood at 37°C then corrected pH, pO_2_, pCO_2_, iCa and HCO_3^−^_, for body temperature once this information was entered. Blood lactate was determined using a portable Lactate Plus™ analyser (Nova Biomedical, Waltham, Massachusetts, 02454 USA).

## Results

**Figure 1:**
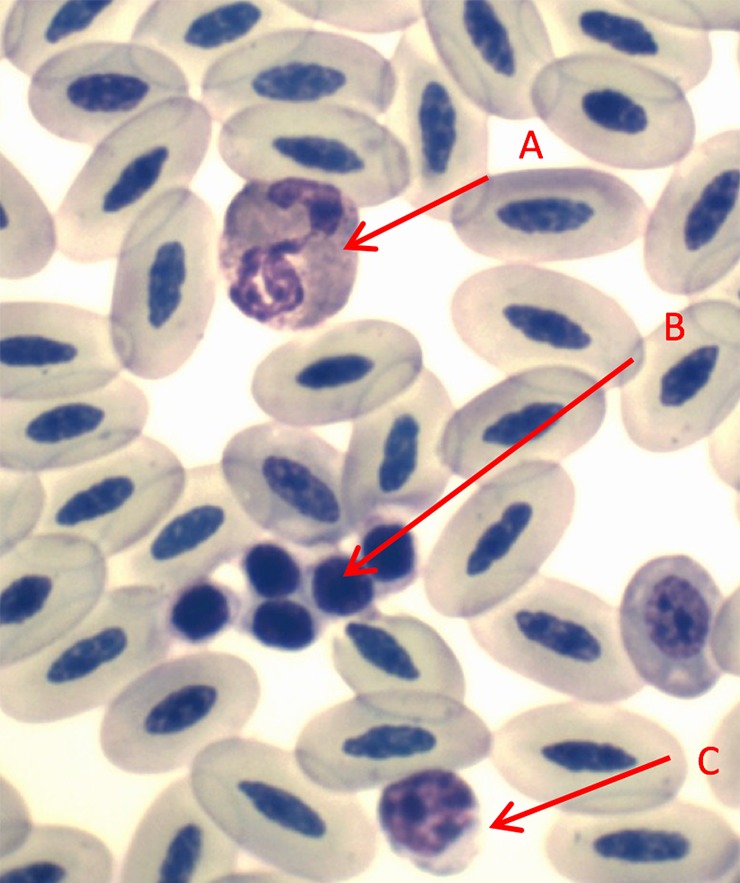
This high magnification photomicrograph illustrates three different white blood cells (A = eosinophil; B = thrombocyte; C = lymphocyte) amongst numerous red blood cells. Eosinophils have muddy pink granules and a lobed nucleus, whereas lymphocytes have a distinctive round nucleus with clumped chromatin and clear blue cytoplasm. Diff Quik stain, 1000×.

**Figure 2:**
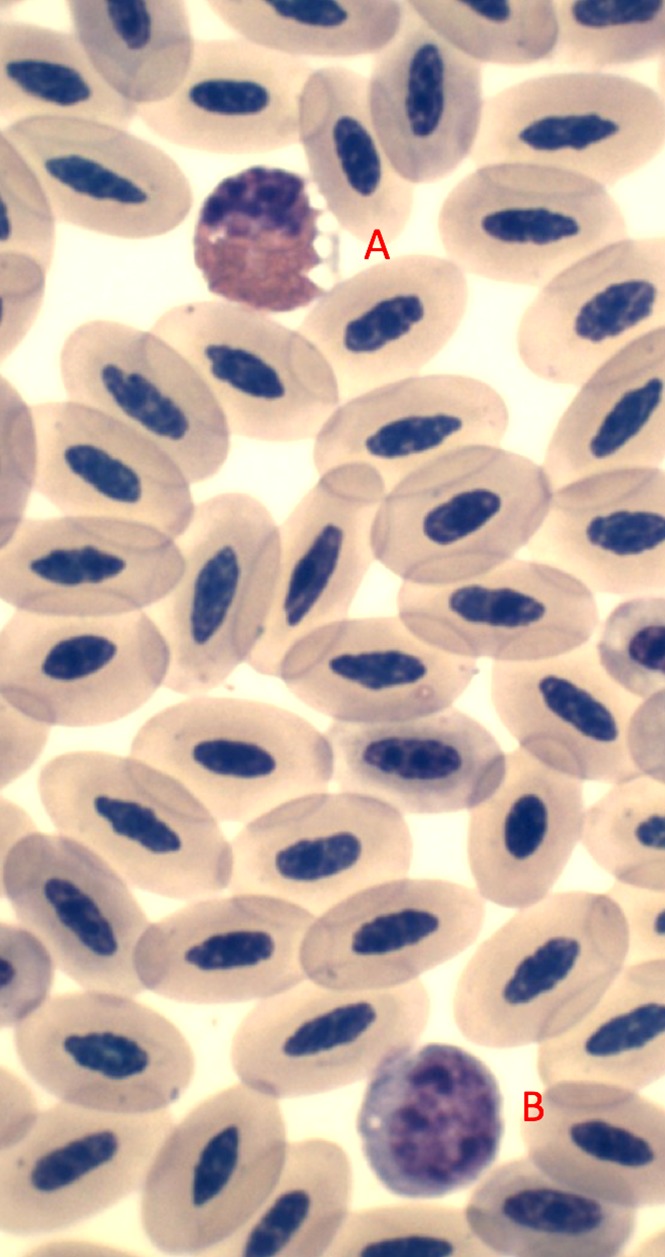
This high magnification photomicrograph illustrates two different white blood cells (A = heterophil; B = lymphocyte) amongst numerous red blood cells. Heterophils showed pink round granules and big vacuoles, whereas lymphocytes have a distinctive round nucleus with clumped chromatin and clear blue cytoplasm. The heterophil and lymphocyte are surrounded by erythrocytes. Diff Quik stain, 1000×.

**Figure 3:**
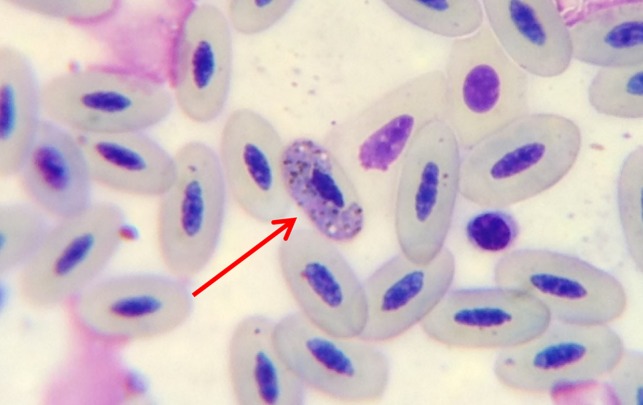
This high magnification photomicrograph illustrates a *Haemoproteus* sp. gametocyte inside a red blood cell. Diff Quik stain, 1000×.

Table 1 summarizes all morphological and physiological measurements as well as the time to obtain blood samples (from capture). Table 2 contains all of the blood chemistry and haematology values. Eosinophil, heterophil and lymphocyte cell types are identified (Figs 1 and 2) and quantified in Table 2. *Haemproteus*-like parasites were found in 32% of the blood films of Punta Pitt samples (Fig. 3) but none were observed from the North Seymour animals.

**Table 2:** Mean, standard deviation, and range for blood gas, biochemical and haematology values for great frigatebirds (*Frigata minor*); 17 males and eight females (*n* = 25) from Punta Pitt and 18 males and 12 females (*n* = 30) from North Seymour Island

Sites	Parameter	Male (*n* = 17)	Female (*n* = 8)
Punta Pitt (2016)	HCT_I_(%)	37.0 ± 3.0 (31–42)	39.13 ± 4.7 (32–46)
	HCT_M_ (%)	54.5 ± 13.9 (25–75)	56.6 ± 9.4 (45–75)
		(*n* = 13)	(*n* = 7)
	Total protein (g/L)	43.6 ± 8.5 (32–64)	40.8 ± 5.6 (36–52)
		(*n* = 12)	(*n* = 7)
	Haemoglobin (g/dL)	12.6 ± 1.03 (10.5–14.3)	13.30 ± 1.57 (10.9–15.6)
	pH (temp. corr)	7.36 ± 0.076 (7.22–7.51)	7.34 ± 0.08 (7.26–7.47)
	HCO_3^−^_ (mmol/l)	21.3 ± 4.66 (11.8–31.2)	20.72 ± 1.79 (18.4–23.6)
	pCO_2_ (mmHg)	37.39 ± 6.86 (23.2–49.7)	39.71 ± 6.73 (28.4–49)
	TCO_2_ (mmHg)	22.24 ± 4.79 (12–42)	22 ± 1.85 (20–25)
	pO_2_ (mmHg)	51.88 ± 19.69 (37–105)	51 ± 18.86 (32–88)
	Na (mmol/l)	141.24 ± 3.07 (138–148)	142.75 ± 3.49 (138–148)
	K (mmol/l)	3.79 ± 0.57 (3–5.3)	3.98 ± 0.743 (3.3–5.7)
	iCa (mmol/l)	1.08 ± 0.15 (0.61–1.28)	1.13 ± 0.09 (0.92–1.22)
	Glucose (mmol/l)	269.65 ± 30.76 (202–311)	281.75 ± 26.1 (239–311)
	Lactate (mmol/l)	3.43 ± 0.95 (2.3–5.6)	2.96 ± 0.94 (1.8–4.2)
	Cell type		
	Heterophil %	29.50 ± 13.13 (12–56.5)	26.25 ± 7.08 (13.5–32)
	Monocyte %	1.32 ± 0.85 (0–3.5)	1.37 ± 0.92 (0.5–3)
	Eosinophil %	31.09 ± 12 (10.5–56)	34.31 ± 11.21 (19–52.5)
	Lymphocyte %	38.06 ± 15.72 (19.5–74)	38.0 ± 14.95 (16–66.5)
	Basophil %	0.029 ± 0.121 (0–0.5)	0.125 ± 0.232 (0–0.5)
	Estimated WBC (x10^9^/l)	3588.24 ± 494.82 (2800–4500)	3812.5 ± 891.92 (2700–5700)

Sites	Parameter	Male (*n* = 18)	Female (*n* = 12)
North Seymour (2017)	HCT_I_(%)	36.44 ± 2.35 (32–40)	36.91 ± 3.8 (28–42)
	Total Protein (g/l)	49.2 ± 11.9 (13–62)	48.5 ± 9.2 (28–62)
	Haemoglobin (g/dl)	12.39 ± 0.8 (10–13)	12.55 ± 1.3 (9.5–14.3)
	Creatinine	0.61 ± 0.27 (0.2–1)	0.44 ± 0.35 (0.2–1.1)
	Urea Nitrogen	5.37 ± 1.9 (3–9)	5 ± 1 (4–6)
	TCO_2_ (mmHg)	22.16 ± 3.18 (15–27)	22.83 ± 4.68 (16–33)
	Anion Gap	13.05 ± 3.01 (5–18)	11.83 ± 4.34 (2–16)
	Cl (mmol/l)	112.16 ± 4.64 (100–120)	112.66 ± 5.85 (100–123)
	Na (mmol/l)	142.44 ± 3.2 (134–148)	142.41 ± 3.39 (134–146)
	K (mmol/l)	3.77 ± 0.46 (3–4)	3.55 ± 0.44 (2–4)
	iCa (mmol/l)	1.13 ± 0.13 (0.7–1.3)	1.06 ± 0.22 (0.57–1.29)
	Glucose (mmol/l)	253.44 ± 22.42 (205–298)	267.25 ± 47.07 (171–330)
	Lactate (mmol/l)	3.31 ± 1 (1–4)	3.98 ± 1.09 (2–7)
	Cell type		
	Heterophil %	42.38 ± 4.16 (34–51)	41.05 ± 3.03 (36–45)
	Monocyte %	0.36 ± 0.61 (0–2)	0.25 ± 0.48 (0–1)
	Eosinophil %	20.88 ± 4.74 (8–29)	21.4 ± 4.12 (15–27)
	Lymphocyte %	36.36 ± 4.56 (30–46)	37.3 ± 3.54 (33–43)
	Basophil %	0 ± 0	0 ± 0

_I_—iSTAT.

_M_—Manual.

Eosinophils were the most predominant granulocyte for Punta Pitt’s colony and heterophils for North Seymour’s. The cell morphology appeared to be normal although the heterophils showed distinctive vacuolization. Basophils were rarely observed. The red blood cell and thrombocyte morphology appeared normal.

## Discussion

This study reports an in depth array of morphometric, vital, blood biochemical and haematological parameters from Galápagos great frigatebirds. In general the data were consistent with values found in other frigatebirds populations. The female birds were consistently larger than the male birds, both in extremity length and body weight (Table 1). Body temperature and respiratory rate were fairly comparable between species and locations. Both populations of female birds had slower heart rates, possibly related to their larger body size, a trend consistent among avian species (Calder, 1968; Lindstedt & Calder, 1976, 1981).

The time interval between bird capture and blood collection varied slightly but not significantly (Table 1). The blood sampling times were slightly longer at Punta Pitt (average of about 7 min) compared to North Seymour (average of ~5 min). This is most likely due to the fact that the field team was more experienced and efficient in 2017, the year of the North Seymour field work. Efforts were made to minimize this variability while keeping the animal’s welfare a priority. In addition, all of the blood parameters, with the possible exception of glucose, would remain fairly stable during the sampling time intervals recorded in this study.

Some of the San Cristóbal and North Seymour haematology values are comparable to data from great frigatebirds on the Galápagos island of Genovesa (Padilla *et al.*, 2006) and in Hawaii (Work, 1996). The differential WBC proportions from the females of the North Seymour colony resemble those of the Hawaiian female great frigatebirds (Work, 1996), with heterophils being the dominant cells (58%; 5.73 10^3^/μl) followed by the lymphocytes (33%; 3.26 10^3^/μl) and eosinophils (6%; 0.57 10^3^/μl). Lymphocytes were the prevalent white cells in the Punta Pitt colony 38% ±15, the same as the Genovesa great frigatebirds 40% ±12 (Padilla *et al.*, 2006). Basophil morphology appeared consistent with basophils in the Pelecaniformes (Clark *et al.* 2009). The leucocyte profiles of the two populations in this study were quite different. This may be significant, since in birds, the heterophils to lymphocyte ratio (H:L) can be useful as an indicator environmental stressors since heterophil numbers rise in the presence of stress and/or infectious agents (Campbell, 1995). In this study, the *H*:*L* ratio of the Punta Pitt colony was 0.69 in females and 0.77 in males. The North Seymour colony had a much higher ratio (1.1 in females and 1.16 in males). These results are primarily due to the percentage differences in heterophil numbers of the two colonies (Table 2). The only potential difference between the two populations, other than geography, is that the Punta Pitt animals experience very little human interaction (tourists are not allowed to go ashore at Punta Pitt) while there is a tourist path on North Seymour in close proximity to the nesting sites.

The haematocrits (HCT%) for the San Cristóbal and North Seymour birds were lower than those reported for Hawaiian (Work, 1996) and Genovesa birds (Padilla *et al.* 2006). This is likely due to the fact that the iSTAT has been shown to produce haematocrits lower than manually (via centrifuge) determined values (Wolf *et al.*, 2008; Rettenmund *et al.*, 2014). Glucose levels were generally comparable between all sites and islands with the values in our study being 10–20% higher than those of Hawaii and Genovesa (Work, 1006; Padilla *et al.*, 2006). It is important to mention that the iSTAT unit was designed as a fast and efficient way to determine blood gas and chemistry parameters in humans. For this reason, future work should devote effort to validate these results with methods that are more commonly used for avian work in brick and mortar laboratories (Steinmetz *et al.*, 2007; Harter *et al.*, 2015). Due to logistical, financial and biological sample export and transport challenges, this was not done.

We sampled the birds in this study during the breeding season while they were on nests. It is entirely possible, if not likely, that blood values would differ during the non-breeding season (Martin *et al.*, 1975; Newman *et al.*, 1997; Navarro *et al.*, 2009). For example, we know that luteinizing hormone and testosterone levels are higher in males that are courting and displaying versus those that are not (Chastel *et al.*, 2005). Similar differences might exist for other blood plasma components.

Threats to seabird health, including the introduction of disease, are likely to increase on the Galápagos as a consequence of human population growth and increased mobility between continental Ecuador and the Archipelago and among islands (Walsh and Mena, 2016). Tourism in the Galápagos is increasing each year and there is no indication this trend will abate in the near future (Walsh and Mena, 2016). With the increased number of tourists comes increased human pressure on popular wildlife sites (e.g. seabird rookeries), increased demand on resources by a growing human population to support the tourist industry, and an overall increased risk for a human-initiated environmental misstep or disaster. The more we know about the vital parameters of healthy animals the better our ability to monitor and make changes, if necessary, to human practices and actions. This health assessment expands the foundation for future work in this species and other Galápagos seabirds.
